# Diagnostic approach of eosinophilic spongiosis^☆^^☆☆^

**DOI:** 10.1016/j.abd.2019.02.002

**Published:** 2019-10-26

**Authors:** Karina Lopes Morais, Denise Miyamoto, Celina Wakisaka Maruta, Valéria Aoki

**Affiliations:** a Postgraduate Program in Dermatology, Department of Dermatology, Hospital das Clínicas, Faculdade de Medicina, Universidade de São Paulo, São Paulo, SP, Brazil; b Department of Dermatology, Hospital das Clínicas, Faculdade de Medicina, Universidade de São Paulo, São Paulo, SP, Brazil

**Keywords:** Diagnosis, differential, Eosinophils, Pemphigoid, bullous, Pemphigus, Skin diseases, vesiculobullous

## Abstract

Eosinophilic spongiosis is a histological feature shared by some distinct inflammatory disorders, and is characterized by the presence of intraepidermal eosinophils associated with spongiosis. Most often, isolated eosinophilic spongiosis indicates the early stages of a subjacent autoimmune bullous dermatosis, such as the pemphigus group and bullous pemphigoid. Herein, the main causes of eosinophilic spongiosis are discussed, as well as the supplementary investigation needed to elucidate its etiology.

## Introduction

Eosinophilic spongiosis (ES) is defined by the presence of intraepidermal eosinophils in spongiotic zones, whether or not associated with intraepidermal vesication.^1^ ES is a histopathological feature shared by different disorders, such as the early stages of autoimmune bullous dermatosis (AIBD), eczema, and drug reaction, thus representing a diagnostic challenge.^1^ A careful clinicopathological correlation is recommended in order to establish the etiology of ES.

### ES in AIBD

Spongiosis associated with epidermal eosinophilic infiltration was first described in 1968 as a pre-acantholytic inflammatory change observed in both pemphigus vulgaris and foliaceus, often preceding its typical clinical and histological presentation.^2^ ES may be the sole alteration or may appear adjacent to acantholytic areas.

**Figure 1:**
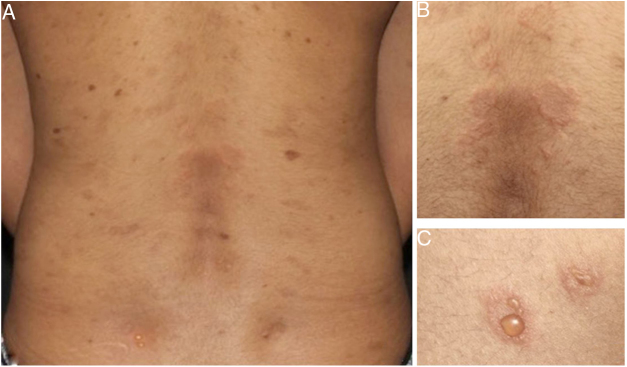
Clinical presentation of pemphigus herpetiformis (A). Annular urticarial plaques (B) and peripheral vesicles (C) in herpetiformis pattern on posterior trunk.

**Figure 2:**
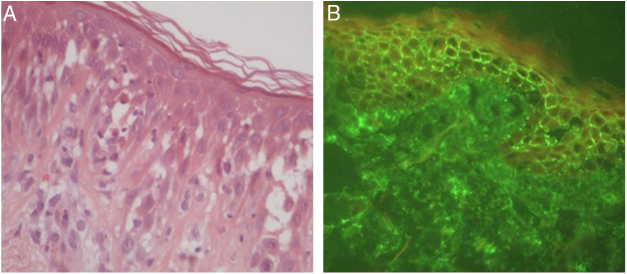
Pemphigus herpetiformis. (A) Eosinophilic spongiosis, without prominent acantholysis (Hematoxylin & eosin, x400). (B) Direct immunofluorescence with linear, intercellular, and intraepithelial IgG deposits.

Later, ES was considered a relevant histological aspect of pemphigus herpetiformis (PH), an unusual clinical variant of pemphigus.^3^ PH clinically resembles dermatitis herpetiformis, and is characterized by pruritic urticarial erythema with vesicobullous eruption in about 50% of cases (Fig. 1). Acantholysis may not be evident by histopathology, but ES is invariably present; immunofluorescence studies with intraepidermal intercellular deposits confirm the diagnosis of PH^3^, ^4^ (Fig. 2).

There were 27 cases of PH diagnosed at the Department of Dermatology of Hospital das Clínicas – University of São Paulo Medical School in the last 15 years, corresponding to 5% of all pemphigus patients under follow-up at this clinic. Among them, ES was the main anatomopathological feature (present in 100% of the cases) and was considered by the authors as a mandatory criterion for PH, with or without concomitant evidence of acantholysis.

ES was also described as the initial histological finding in one case of paraneoplastic pemphigus.^5^ Additionally, pemphigus vegetans may display ES commonly associated with supra-basal acantholysis and epidermal hyperplasia.^1^, ^6^

**Figure 3:**
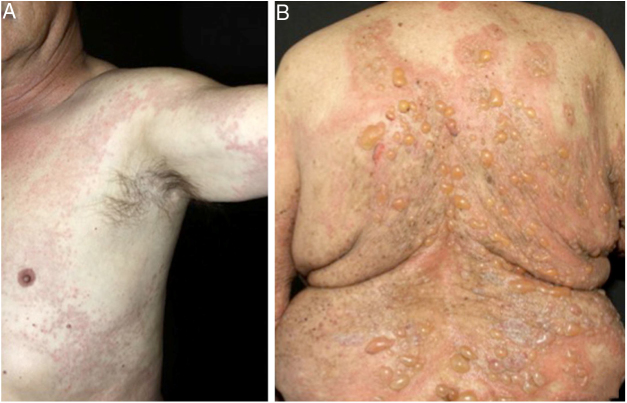
Urticarial (A) and bullous (B) phases of bullous pemphigoid.

**Figure 4:**
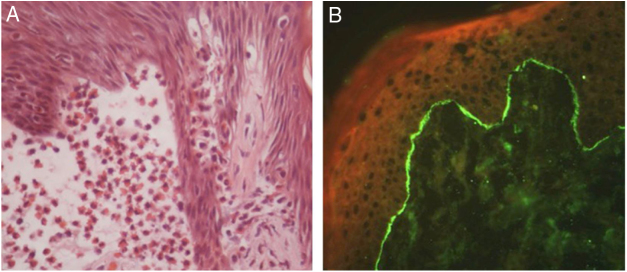
Bullous pemphigoid. (A) Focal eosinophilic spongiosis adjacent to subepidermal clefting (Hematoxylin & eosin, x400). (B) Direct immunofluorescence with linear deposits of IgG in the basement membrane zone.

In bullous pemphigoid (BP), ES is a prominent feature, even in the absence of adjacent subepidermal detachment. It is especially observed during the pre-bullous phase, when urticarial lesions, eczema, or even isolated pruritus prevail (Figure 3, Figure 4).^6^, ^7^ This finding may not be fortuitous, as previous studies demonstrated the role of eosinophils in the pathogenesis of BP. It seems that the release of toxic proteins by eosinophils can contribute to blister formation.^7^ It is hypothesized that chemokines released by keratinocytes after epidermal damage induce eosinophilic migration into epidermis in BP, including IL-8 and eotaxin.^7^

Ruiz et al. observed that among 150 patients with ES, 24% had an underlying AIBD, emphasizing BP as the main cause.^8^ Mucous membrane pemphigoid and pemphigoid gestationis are less frequently associated with ES. During pregnancy, the occurrence of ES in urticarial lesions may support the diagnosis of pemphigoid gestationis and help to distinguish it from polymorphic eruption of pregnancy.^6^

### Other differential diagnoses

Even though ES is traditionally associated with AIBD, it has been accepted as a consistent histological feature of other inflammatory skin disorders, notably spongiotic dermatitis.^6^ Although lymphocytes are the main inflammatory cells, ES can occur adjacent to other epidermal alterations in eczema, such as in contact, atopic, or nummular dermatitis.^6^, ^9^ Ruiz et al. found that most patients with isolated ES had either eczematous dermatitis or AIBD without concomitant vesicles or blisters.^8^ In such cases, immunofluorescence studies are required to distinguish both disorders.^1^

Arthropod bite reactions, urticaria, drug reactions, and scabies represent other causes of ES.^1^, ^6^, ^9^ Prominent dermal edema and mixed inflammatory infiltrate are classically seen in insect bite reactions and urticarial lesions.^1^, ^6^ Nevertheless, the urticarial phase of an AIBD must be excluded. In scabies, the presence of the mite in stratum corneum may confirm the diagnosis.^6^ Id reactions secondary to fungal or other infections may also cause ES.^8^

The vesicular phase of incontinentia pigmenti, a rare X-linked dermatosis, may also exhibit ES along with dyskeratotic keratinocytes, and has distinctive histopathological features.^1^, ^6^ ES is seldom observed in lichen sclerosus, polycythemia vera, porokeratosis, Meyerson's nevi, Still's disease, and Wells syndrome.^1^, ^6^, ^9^ Additional histopathological alterations provide more specific evidence to support the diagnosis. In eosinophilic follicular pustular folliculitis (Ofuji folliculitis), ES is found in the hair infundibulum and sebaceous duct.^1^, ^9^

### Diagnostic workup

**Figure 5:**
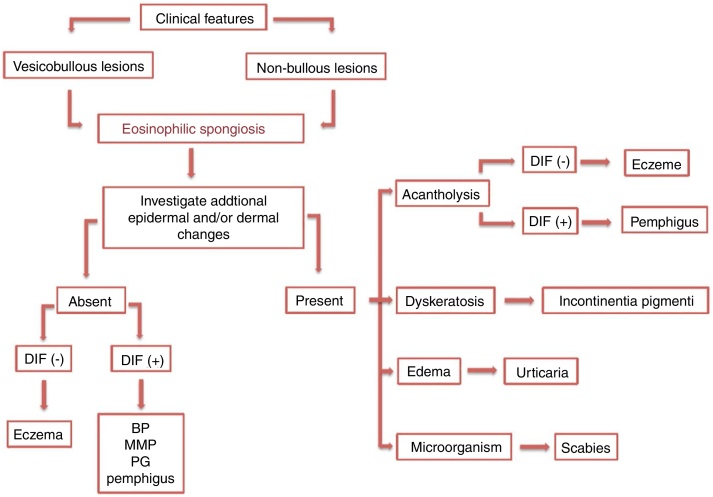
Diagnostic management of eosinophilic spongiosis. DIF, direct immunofluorescence; (−), negative; (+), positive; BP, bullous pemphigoid; MMP, mucous membrane pemphigoid; PG, pemphigoid gestationis.

ES cases with specific clinical and histological features may provide evidence to an accurate diagnosis. In contrast, those with unusual clinical presentation and/or without additional histopathological alterations may represent a diagnostic challenge. (Fig.5)

A careful analytic approach and clinicopathological correlation are often necessary for etiological elucidation. This includes the evaluation of associated epidermal and dermal alterations from several histological serial sections. Acantholysis, dyskeratosis, intense dermal edema, inflammatory infiltrate, basement membrane zone alterations, and the presence of microorganisms may be observed and aid in the diagnosis.

The diagnosis of early AIBD requires ancillary testing. Immunofluorescence (IF) studies are the gold standard for the diagnosis of AIBD to demonstrate the presence of autoantibodies against intra-epidermal or basement membrane zone antigens *in vivo*.^10^ Direct IF detects tissue-bound autoantibodies, whereas indirect IF quantifies circulating autoantibodies. Deposits of fluorescent antibodies are detected in all pemphigus and BP patients, and may be determinant in atypical cases.^10^

## Final considerations

It is recommended to consider AIBD as a differential diagnosis of ES. In this group, special attention must be given to BP and PH due to their higher association with ES. Concomitant clinical and histological features may lead to the correct diagnosis. However, when ES is the main or single histological abnormality on a skin biopsy, active investigation for specific features and immunofluorescence analysis are often necessary, and must be repeated in case of initial negativity.

Concerning the etiology of ES, keratinocyte signaling may play a role in the induction of epidermal eosinophilic infiltration. However, the reason why it occurs in such different disorders remains unknown and has not been studied yet. Future research is still necessary and will be essential to elucidate these pathogenic questions and contribute to the discovery of new therapeutic targets.

## Financial support

The present study received financial support from FUNADERSP (Fundo de Apoio ao Dermatologista de São Paulo), São Paulo, Brazil.

## Author's contributions

Karina Lopes Morais: Approval of the final version of the manuscript; conception and planning of the study; elaboration and writing of the manuscript; obtaining, analyzing and interpreting the data; effective participation in research orientation; critical review of the literature; critical review of the manuscript.

Denise Miyamoto: Approval of the final version of the manuscript; conception and planning of the study; elaboration and writing of the manuscript; effective participation in research orientation; intellectual participation in propaedeutic and/or therapeutic conduct of the cases studied; critical review of the manuscript.

Celina Wakisaka Maruta: Approval of the final version of the manuscript; conception and planning of the study; effective participation in research orientation; intellectual participation in propaedeutic and/or therapeutic conduct of the cases studied; critical review of the manuscript.

Valéria Aoki: Approval of the final version of the manuscript; conception and planning of the study; effective participation in research orientation; intellectual participation in propaedeutic and/or therapeutic conduct of the cases studied; critical review of the manuscript.

## Conflicts of interest

None declared.
